# THE IMPACT OF PERCEIVED IMPORTANCE AND RECENT CHANGES ON PREPARATION FOR OLD AGE

**DOI:** 10.1093/geroni/igz038.2749

**Published:** 2019-11-08

**Authors:** Jeongsoo Park, Thomas M Hess

**Affiliations:** North Carolina State University, Raleigh, North Carolina, United States

## Abstract

This study focused on identifying factors that motivate individuals to make provisions for their old age. We hypothesized that the motivation to make preparations would be associated with the importance attached to a specific domain of functioning along with the extent to which the individual experienced recent change within that domain. We examined how perceived changes in five different domains of functioning (social relationships, leisure, finances, work, health) during the last four years and the importance attached to each domain affected preparation for old age. Participants aged 35 to 85 (Germany: N=811; Hong Kong: N=482; US: N=515) were part of the Aging as Future Study. Across domains, perceptions of positive change and high importance were related to more engagement in preparations, as was being older. The strength of these effects as well as moderating influences varied across domains and cultures, reflecting potential differences in values and social/cultural supports.

